# Production of virus-like particles of porcine circovirus 2 in baculovirus expression system and its application for antibody detection

**DOI:** 10.1186/s12917-023-03648-7

**Published:** 2023-07-19

**Authors:** Yanwei Li, Pingping Yu, Yaxuan Bao, Yuwen Ren, Shaowei Zhao, Xuexian Zhang

**Affiliations:** Beijing Kemufeng Biopharmaceutical Co., Ltd, No.25 Xiangrui Street Daxing District, Beijing, 102600 China

**Keywords:** Porcine circovirus 2, Virus-like particles, Indirect ELISA

## Abstract

**Background:**

Porcine circovirus 2 (PCV-2) is one of the pathogens that leads to a growing and persistent threat in pigs. Thus, the development of serological detection methods for PCV-2 is of great necessity for clinical diagnosis as well as epidemiological investigations. This study aimed to establish an indirect enzyme-linked immunosorbent assay (ELISA) to examine antibodies against PCV-2 based on virus-like particles (VLPs).

**Results:**

Recombinant PCV-2 Cap protein was expressed in the baculovirus-insect cells system and PCV-2 VLPs were observed over transmission electron microscopy (TEM). The PCV-2 VLPs were shown to have good immunogenicity in mice and stimulated a high level of PCV-2 antibody titers. Using PCV-2 VLPs as coating antigen, the indirect ELISA can detect PCV-2 antibodies in animals with diagnostic sensitivity and specificity of 98.33% and 93.33% compared to immunofluorescence assay (IFA), respectively. The intra- and inter-assay coefficient variations (CVs) were < 10% in a batch, and < 15% in different batches, indicating good repeatability. There was no cross-reaction of this ELISA with antibodies against other porcine viruses. A total of 170 serum samples collected from different pig farms in China were tested for PCV-2 antibodies, and 151 (88.8%) samples were PCV-2 antibody positive.

**Conclusion:**

Our findings suggest that this ELISA was rapid, specific, and reproducible and can be used for large-scale serological investigations of PCV-2 antibodies in pigs.

**Supplementary Information:**

The online version contains supplementary material available at 10.1186/s12917-023-03648-7.

## Introduction

Porcine circovirus (PCV), a virus in *Circoviridae* family, contains four distinct types, porcine circovirus 1 (PCV-1), 2 (PCV-2), 3 (PCV-3) and 4 (PCV-4) [1–4]. PCV-1 was initially identified as a contaminant in a culture of the porcine kidney cell line (PK-15) [5] and nonpathogenic for pigs [6]. The newly discovered PCV-3 was first detected in a U.S. sow farm (North Carolina) experiencing porcine dermatitis and nephropathy syndrome (PDNS) and reproductive failure in 2015 [3]. The capsid and replicase proteins of PCV-3 are only 37% and 55% identical to PCV-2. Current understanding of PCV-3 pathogenesis is very limited [7]. In April 2019, a new porcine circovirus 4 with a distinct relationship to other circoviruses was identified in several pigs with severe clinical disease in Hunan province, China [8]. PCV-4 genome has 43.2-51.5% of identities with other PCV genomes. PCV-3 and PCV-4 are relatively new discovery and more research is necessary to determine their possible clinical pathogenic effects [9]. Researchers believe that PCV-2 is the main etiologic agent for a multifactorial clinical disease (porcine circovirus-associated disease, PCVAD), causing major economic losses in the swine industry [10–12]. In addition, the coinfection of PCV-2 and other pathogens, including porcine epidemic diarrhea virus (PEDV) and porcine reproductive and respiratory syndrome virus (PRRSV), increases the clinical severity of PCVAD, leading to a great challenge in preventing the transmission of PCV-2 [13].

PCV-2 antibody detection can reflect the infection of the virus in pig population. At present, common methods for PCV-2 antibody detection include indirect immunofluorescent assay (IFA), immunoperoxidase monolayer assay (IPMA) and enzyme-linked immunosorbent assay (ELISA) [14–17]. Although IFA and IPMA are reliable and accurate for antibody detection, these methods can be labor-intensive and time-consuming, and are associated with the risk of virus contamination. In contrast, ELISAs can avoid these problems and perform large-scale diagnostics. It is well known that the whole virus is currently used as the coating antigens in ELISA systems for the detection of serum antibodies. However, PCV-2 culture is a very arduous procedure, and it is difficult to get a large amount of PCV-2 particles for coating antigens [18]. Furthermore, the inactivated PCV-2 viruses are poorly bind to ELISA plates, affecting their suitability for indirect ELISAs. Therefore, it is critical to overcome these limitations and explore new methods to make the coating antigens for the development of serological diagnostic techniques.

PCV-2 genome includes two main open reading frames, ORF1 and ORF2. ORF1 encodes Rep protein, which is indispensable for initiation of viral replication [19, 20]. ORF2 encodes a sole structural protein, Cap protein, which is the major PCV-2 immunogen [21]. Currently, PCV-2 Cap protein prepared from bacteria has been widely used for research or commercial purposes with the low cost of antigen protein production [22–24]. It is worth noting that PCV-2 Cap monomers can assemble into virus-like particles (VLP), whose structure is the similar to the native virus particles, and have recently been touted as next-generation subunit vaccine candidates [25, 26]. In addition, recombinant protein can reduce its cross-reactivity and thus decrease false positives compared with the whole viruses [27, 28]. Therefore, PCV-2 VLPs may be better to serve as the coating antigens for ELISA systems.

In the present study, we developed an indirect ELISA for PCV-2 antibody detection using PCV-2 VLPs as coating antigen, which obtained from recombinant baculovirus expression system. The sensitivity and specificity of this ELISA assay were characterized. The prevalence of PCV-2 infection in clinical pig samples using this I-ELISA was investigated. Our findings indicate that PCV-2 VLP-based ELISA is highly specific, sensitive, and reproducible. This indirect ELISA may be a valuable tool for monitoring the prevalence of PCV-2 infection.

## Materials and methods

### Serum samples

PCV-2-positive serum was prepared as described by Huang et al. [29]. PCV-2-negative serum was collected from specific-pathogen-free (SPF) piglets which were obtained from the Harbin Veterinary Research Institute (HVRI) of the Chinese Academy of Agricultural Sciences (CAAS, Harbin, China). Other forty negative serum samples used for ELISA cut-off value determination were also collected from SPF piglets.

Blood was collected in BD Vacutainer® SST™ blood collection tubes (BD, Franklin Lakes, NJ) by venipuncture of the anterior vena cava as it exits cranial to the thoracic inlet. The blood was allowed to stand at room temperature for at least 20 min before centrifuging at 3,000 × g for 10 min in serum separator tubes. The serum samples were transferred to 5-mL snap cap tubes (Fisher Scientific, Waltham MA) and stored at -80℃.

One hundred and twenty clinical serum samples stored at Beijing Kemufeng Biopharmaceutical Co., Ltd were tested by IFA and the established PCV-2 indirect ELISA simultaneously.

### Expression and purification of recombinant Cap protein in the baculovirus expression system

The ORF2 sequences of PCV-2b (NCBI accession no. AY099500.1) were synthesized by optimizing the codons for expression in insect cells. By using the *EcoR*I and *Xho*I cutting sites, PCV-2 ORF2 gene was cloned into pFastBac-1 (Invitrogen, USA), recombinant plasmid pFastBac1-ORF2 was then transformed into DH10Bac E. coli (Thermo Fisher Scientific, Waltham, MA, USA). The transformed DH10Bac bacteria were cultivated in LB agar plates containing gentamicin, tetracycline, kanamycin, and X-gal/IPTG, and white colonies were chosen and confirmed to be recombinant bacmid. Spodoptera frugiperda (Sf9) insect cells (Invitrogen, USA) were cultured at 27 °C in SF-900 II SFM media (Gibco, Grand Island, NY, USA), recombinant bacmid DNAs (2 μg) were used for transfection along with 10 μl of Cellfectin II reagent (Invitrogen, USA) in 1 mL of SF-900 II SFM. Transfected cells were incubated for cytopathic development.

Sf9 and Hi5 cells (2 × 10^6^ cells) were cultured in Insect-XPRESS™ (Lonza, Basel, Switzerland) media and infected with PCV-2 recombinant baculoviruses at a multiplicity of infection (MOI) of 1 and cultured at 27℃ with 120 rpm of shaking for 72 h to produce PCV-2 cap proteins. PCV-2 Cap proteins were purified on ÄKTA pure (AKTA, GE-Healthcare Life Sciences, USA) using a 5-mL SP HP column (Genscript, Nanjing, China), The quantity of the purified PCV-2 VLPs was quantified using a Bicinchoninic Acid Assay Kit (Beyotime, Shanghai, China), and then evaluated using SDS-PAGE.

### SDS-PAGE and Western blot

Recombinant PCV-2 Cap protein (10 μg) was resolved on a 12% sodium dodecyl sulfate-polyacrylamide gel electrophoresis (SDS-PAGE) gel and transferred to polyvinylidene (PVDF) membranes. The transferred nitrocellulose blot was blocked with 5% skim milk (BD Pharmingen, San Diego, CA, USA) in PBST [phosphate buffer solution (PBS) containing 0.1% Tween-20] at 4 °C overnight. After being washed with PBST solution three times, the membrane was incubated with PCV-2 positive serum (1/1000 dilution in PBS) at 4 °C overnight, and hybridized with HRP-conjugated goat anti-pig IgG (Solarbio, Beijing, China) (1/2000 dilution in PBST) for 1 h at room temperature. At last, the detection was performed using Enhanced Chemiluminescence Plus Kit (Bio-Rad Clarity Western ECL; Bio-Rad Laboratories Inc.).

### Electron microscopy

The purified VLPs were coated onto a formvar carbon film with 400-mesh formvar copper grids for 1 min at room temperature, gently air-dried, and negatively stained with 2% phosphotungstic acid, pH 7.0, and examined by using a transmission electron microscope (H7650, HITACHI, Japan).

### Indirect immunofluorescence assay (IFA)

IFA assay was performed using a modification of previously established procedures [30]. Briefly, confluent monolayers of PK-15 cells infected with PCV-2 strain LG (MOI = 0.01) which is kindly gifted from Dr. Wei (HVRI, Chinese Academy of Agricultural Sciences) were fixed in 80% ice acetone at 4℃ for 30 min. After being washed three times with PBS (0.01 mol, pH7.2), the plates were kept at -20℃. The serum samples (n = 120) were introduced to the PCV-2- and mock-infected PK-15 cells, respectively, 50 μL/well, and diluted 1:50 with PBS (0.01 mol, pH7.2), before being incubated at 37℃ for 60 min. The anti-PCV-2 serum and SPF serum were made as a positive and negative control, respectively. After three times washing, 50 μL of 1:200 dilution of FITC-conjugated goat anti-guinea Pig IgG (Sigma, St. Louis, MO, USA) was added and incubated at 37 °C for 60 min. The plates were washed with PBS and examined under an immunofluorescence microscope (Life Technologies, Gaithersburg, MD, USA).

### Preparation of mouse PCV-2 sera

The BALB/c mice were raised in a temperature-adjustable environment (22 °C ± 1 °C) with six per cage on a 12 h light/12 h dark cycle with free access to food and water. Three female BALB/c mice (aged 6–8 weeks) were intraperitoneally injected with PCV-2 VLPs (50 μg) in Freund’s complete adjuvant (Sigma, St. Louis, MO, USA) in 1:1 ratio (V/V). The identical dose of VLPs in Freund’s incomplete adjuvant (Sigma, St. Louis, MO, USA) was delivered intraperitoneally twice at interval of two weeks. Mice blood was drawn two weeks after the last booster, and the sera were prepared and kept at -80℃ before analysis.

This study was performed in accordance with the recommendations in the Guide for the Care and Use of Laboratory Animals of Beijing Kemufeng Biopharmaceutical Co., Ltd (approval number DW202003-010).

### Optimization of indirect ELISA working conditions

Standard checkerboard titration techniques were used to determine the ideal antigen and serum dilutions [31]. Briefly, 96-well ELISA plates were coated with pure PCV-2 VLPs (0.5, 1.0, 1.5, 2.0, 2.5 to 5 μg/mL) in 100 μL Carbonate buffer solution (CBS, pH 9.6; Corning, NY, USA). Accordingly, the dilution ratios of PCV-2-positive and -negative serum in PBS were 1:50, 1:100, 1:150, and 1:200 (v/v). The ratio of PCV-2-positive to PCV-2-negative serum OD450 (P/N value) was used to find the best dilutions. The best working circumstances were determined to be those that produced the highest P/N value and an OD450 of positive serum that was close to 1.0 [32].

### Establishment of indirect ELISA (I-ELISA)

Purified PCV-2 VLPs (2 μg/mL) were applied to the wells of an ELISA plate at 100 μl/well. After three PBST washes, 300 μL of 5% bovine serum albumin (BSA, Sigma, St. Louis, MO, USA) was added to each well, and the plate was blocked for 1.5 h at 37 °C. 100 μL of diluted serum (1:50) was added after three times of washing and kept at 37 °C for 0.5 h. After three times of PBST washing, 100 μL 1:30,000 HRP-labeled goat anti-pig IgG (Solarbio, Beijing, China) was added and incubated for 0.5 h at 37 °C. 100 μL 3,3,5,5-tetramethylbenzidine (TMB) substrate solution was added and incubated for 15 min at 37 °C. Finally, the reaction was stopped with 50 μL/well of 2 mol sulfuric acid, and the absorbance was measured at 450 nm using a microplate reader (Bio-Rad, Hercules, CA, USA).

### Determination of cut-off value

The cut-off value was established using 40 negative serum samples from SPF piglets. IFA test analysis was used to determine if the serum was PCV-2-positive as above described. The PCV-2 VLP ELISA was performed on all sera in duplicate. The cut-off value of OD450 in the conversion was based on the following formula: (OD450 value of the sample − OD450 value of the negative)/(OD450 value of the positive − OD450 value of the negative). The mean S/P ratio plus three standard deviations (SD) were used to calculate the cut-off ratio.

### Evaluation of the analytical sensitivity and specificity of I-ELISA

I-ELISA was performed using a serial of two-fold dilutions of the PCV-2-positive sera ranging from 1:10 to 1:1280 to determine the sensitivity. The specificity of the I-ELISA was evaluated using the positive sera of porcine parvovirus (PPV), porcine reproductive and respiratory syndrome virus (PRRSV), porcine epidemic diarrhea virus (PEDV), transmissible gastroenteritis coronavirus (TEGV), pseudorabies virus (PRV), and classical swine fever virus (CSFV) with triplicate experiments, and the S/P value was used to characterize whether the samples were positive or negative. A positive control was provided by PCV-2-positive serum, whereas a negative control was provided by PCV-2-negative serum.

### Repeatability of the indirect ELISA

A total of 5 serum samples were chosen to assess the repeatability of this ELISA using a modification of previously established procedures [33]. The coefficient of variation (CV) for each sample was calculated both within and between plates (inter-assay variation and intra-assay variation). The triplicates of each serum sample were examined for inter-assay repeatability using the same batch of pre-coated ELISA plates. Each serum sample was detected by three batches of pre-coated ELISA plates for the intra-assay repeatability. Each test’s mean S/P value and CV were computed.

### Evaluation of the diagnostic sensitivity and specificity of I-ELISA

The diagnostic sensitivity (DSe), diagnostic specificity (DSp), positive predictive value (PPV), and negative predictive value (NPV) of established I-ELISA were all evaluated using a total of 150 field serum samples examined by IFA. The number of true positives (TPs), true negatives (TNs), false positives (FPs), and false negatives (FNs) was assessed in a two-by-two table (FNs). In order to compare ELISA with IFA, the following statistical techniques were used: DSe = TP/(TP + FN), DSp = TN/(TN + FP), PPV = TP/(TP + FP), and NPV = TN/(TN + FN).

### Detection of PCV-2 antibodies by the PCV-2 VLP I-ELISA

A total of 170 pig serum samples were collected from farms in Shandong Province (65 samples), Hunan Province (35 samples) and Henan Province (70 samples) in China. All animals had been vaccinated with an inactivated chimeric PCV-2 vaccine (Sichuan HuaPai Bio-pharmaceutical Co., Ltd., Chengdu, China). The serum samples were tested by the PCV-2 indirect ELISA established in this study. Each sample was tested in triplicates.

### Statistical analysis

All statistical data were analyzed using GraphPad Prism 5.0 (GraphPad Software). Data are expressed as the mean ± SD. The intra- and inter-assay variation was evaluated by the coefficient of variation (CV).

## Results

### Determination of PCV-2 VLPs

**Fig. 1:**
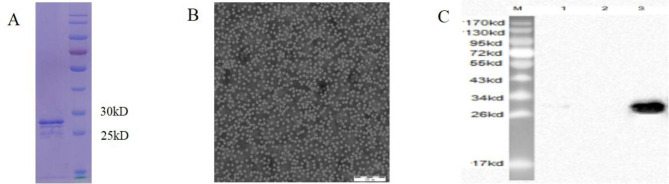
Analysis and purification of PCV-2 Cap in baculovirus-expressed system. (**A**) SDS-PAGE result of the purified PCV-2 Cap. (**B**) TEM images of purified PCV-2 Cap. The bar indicates 200 nm. (**C**) Western blots with swine PCV-2-specific positive serum. M, molecular weight marker; 1, uninfected Sf9 cell lysate; 2, Sf9 cells infected with pFastBac™ Dual plasmid; 3, purified PCV-2 Cap protein

To assess the expression of recombinant PCV-2 Cap in insect cells, SDS-PAGE was performed. An estimated 27 kDa protein was observed (Fig. 1A). Using affinity chromatography, PCV-2 Cap protein was purified and determined. The purified PCV-2 Cap proteins were also examined by immunoblot analysis using a PCV-2-positive serum (Fig. 1C). Furthermore, the assembly of PCV-2 VLPs based on the Cap proteins prepared as above was examined using TEM. A large number of regular spherical particles with ~ 17 nm in diameters were observed under TEM, which was similar to natural PCV-2 virions (Fig. 1B). Therefore, PCV-2 VLPs were successfully obtained from recombinant Cap proteins in insect cells.

### The antigenic specificity of PCV-2 VLPs

**Fig. 2:**
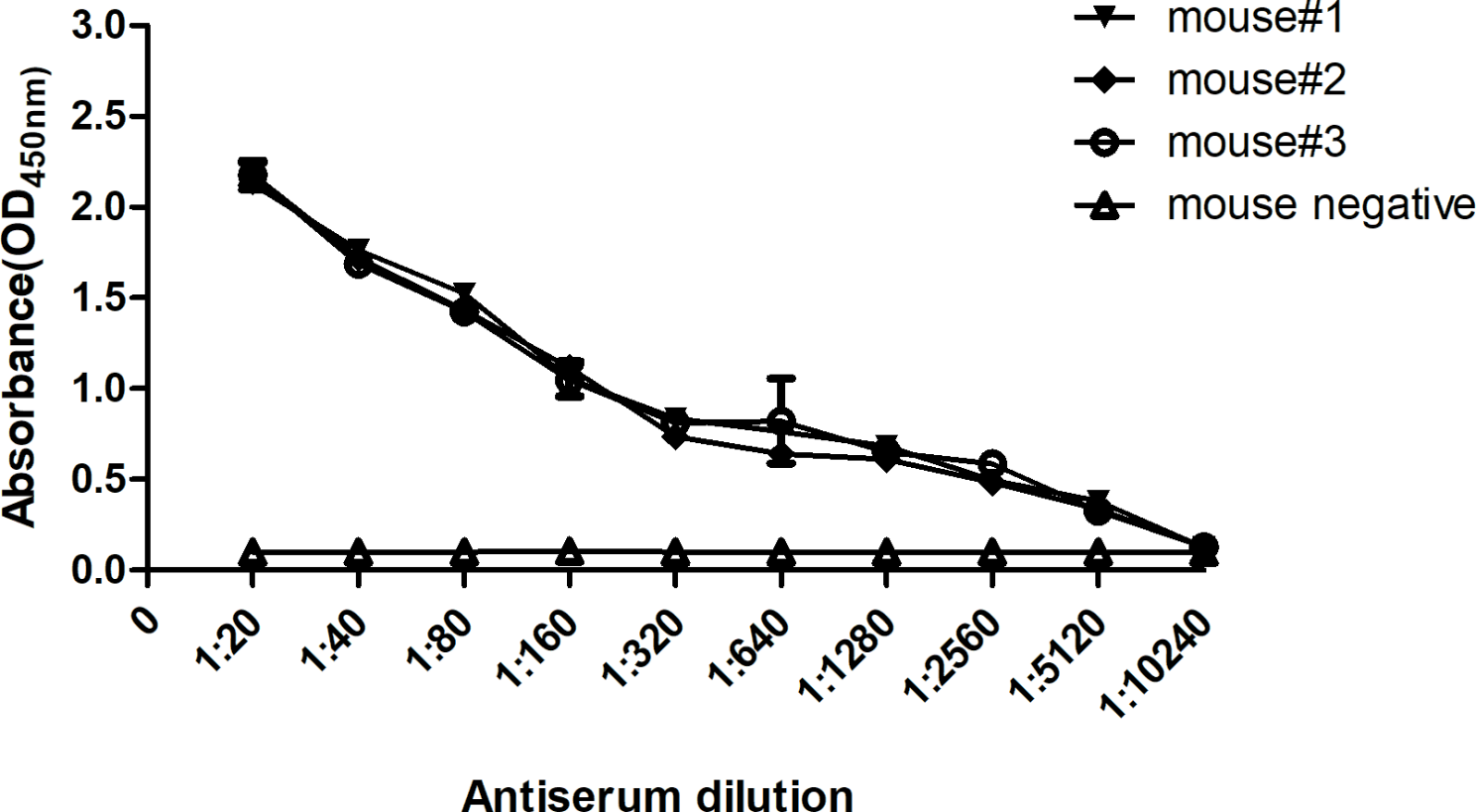
Determination of antibody titers in immunized mice. BALB/c mice were intraperitoneally injected with PCV-2 VLPs, and the sera of mice were prepared after four immunizations with the same dose of VLPs. The antibody titers of the sera were determined by the I-ELISA developed

The antigenic specificity of PCV-2 VLPs was examined for using as the coating antigens in the ELISA. A series of two-fold dilutions of the sera from immunized mice were subjected to this ELISA with PCV-2 VLPs. Specific reactivity was observed compared with control mice (Fig. 2). The result indicated that PCV-2 VLPs were capable of serving as coating antigens to detect PCV-2-specific antibodies.

### Optimization of an indirect ELISA for detection of PCV-2 antibodies

The optimal reaction conditions for the PCV-2 I-ELISA were determined with OD_450_ and P/N values. Using checkerboard titration, the optimal amount of coating antigen PCV-2 VLPs was measured at 200 ng/well, and the optimal serum sample dilution was 1:50 (Table 1). Through optimizing the conditions, this ELISA were set as follows: coating process was 4℃ overnight; blocked with 300 μL 5% bovine serum albumin (BSA) at 37 °C for 90 min; 1:30,000 dilution of HRP-labeled goat anti-pig IgG; visualization with 100 μL TMB at 37 °C for 15 min; and reaction termination with the addition of 50 μl of 2 M sulfuric acid per well.

**Table 1:** Checkerboard titration of the PCV VLPs

	Serum dilution				
		1:20	1:50	1:100	1:200
Concentrations of antigen (μg/mL)	0.5	0.800/0.101	0.704/0.065	0.427/0.054	0.252/0.032
	P/N	7.92	10.83	7.904	7.875
	1.0	0.926/0.111	0.743/0.070	0.463/0.056	0.312/0.035
	P/N	8.34	10.485	8.26	7.0
	1.5	1.031/0.120	0.822/0.072	0.546/0.063	0.335/0.041
	P/N	8.59	11.41	8.67	8.17
	2.0	1.101/0.141	0.853/0.070	0.581/0.057	0.387/0.042
	P/N	7.8	12.19	10.19	9.21
	2.5	1.108/0.149	0.932/0.082	0.630/0.075	0.458/0.047
	P/N	7.44	11.36	8.4	9.7
	5.0	1.232/0.184	0.980/0.100	0.728/0.081	0.541/0.051
	P/N	6.70	9.8	8.98	10.06

### Determination of the I-ELISA cut-off value

**Fig. 3:**
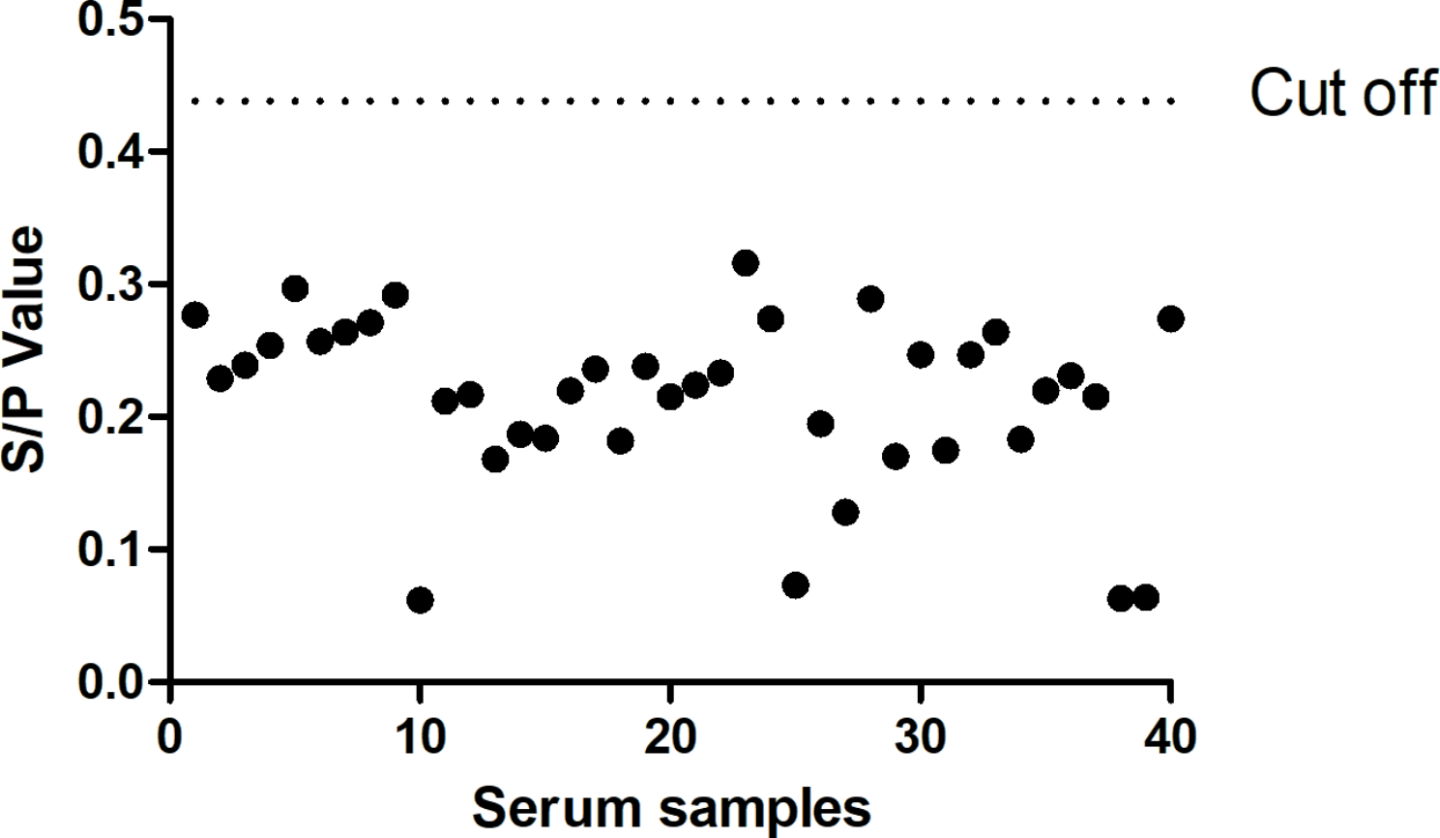
Evaluation of the cut-off value of the indirect ELISA. The black dotted line presents the S/P cut-off value (0.438)

To determine the cut-off value of PCV-2 VLP I-ELISA, forty negative serum samples were tested. After three independent experiments, the mean of the S/P values for these samples by the PCV-2 VLP I-ELISA was 0.114 and standard deviation 0.108. The cut-off value was determined as the mean plus three standard deviations (SD) of negative sera according to a Gaussian population distribution. Therefore, this ELISA threshold was 0.114 + 3 × 0.108 = 0.438, indicating that serum samples were positive as S/P value was ≥ 0.438 and negative less than 0.438 (Fig. 3).

### Evaluation of the analytical sensitivity, specificity and repeatability of I-ELISA

**Fig. 4:**
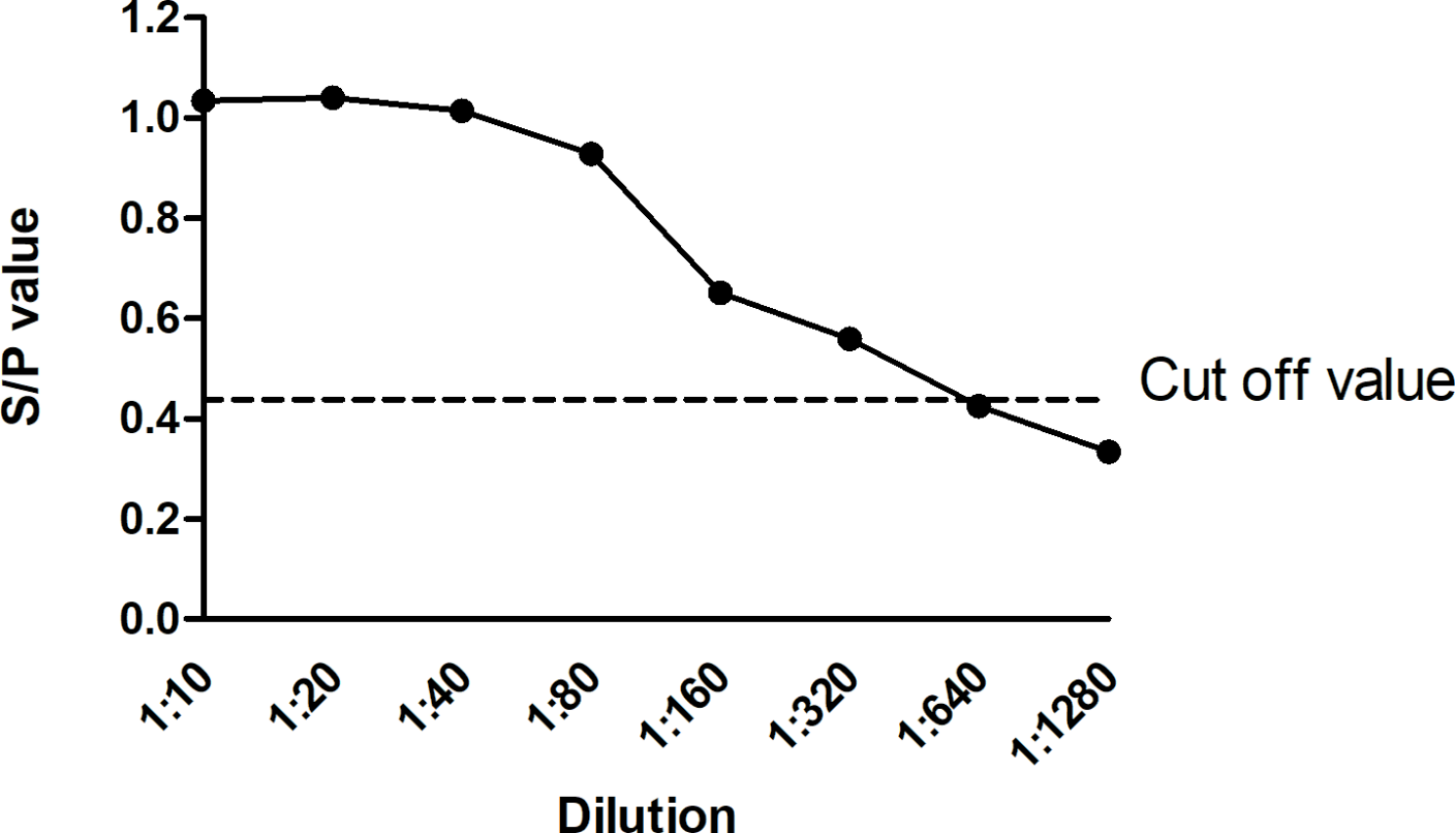
Testing of the sensitivity of the I-ELISA. The PCV-2-positive sera were diluted in serial twofold dilutions from 1:10 to 1:1280, and the diluted serum was tested by I-ELISA.

The analytical sensitivity of the indirect ELISA was assessed using PCV-2 -positive serum samples, where the positive serum was serially two-fold diluted from 1:10 to 1:1280. The highest dilution for anti-PCV-2 serum was 1:320 for the positive pig serum by using the cut-off value of 0.438 (Fig. 4).

The analytical specificity of I-ELISA was evaluated by testing samples that were sero-positive for PPV, PRRSV, PEDV, TEGV, PRV and CSFV. Triplicates of each sample were run on the I-ELISA. As shown in Table 2, no cross-reactivity was observed between the PCV-2 and any of these anti-sera, proving that PCV-2 VLP was specific for PCV-2 antibodies.

**Table 2:** S/P value of PCV2 ELISA for other pathogens

Pathogen	S/P
PPV	0.067 ± 0.053
PRV	0.106 ± 0.042
PRRSV	0.098 ± 0.036
CSFV	0.081 ± 0.037
TGEV	0.077 ± 0.028
PEDV	0.082 ± 0.031

The repeatability of the I-ELISA assay was evaluated by calculating the S/P value of each selected clinical sample. Triplicates of each sample were run on the same batch. The standard deviations (SD) and CVs were then calculated. As shown in Table 3, the intra-assay CV of 5 positive serum samples ranged from 1.8 to 5.7%, the inter-assay CVs for positive serum samples was between 2.0% and 5.3%. Collectively, these data demonstrated that this new I-ELISA was reliability.

**Table 3:** Repeatability test of VLPs-I-ELISA

Sample ID	Intra-batch repeatability			Inter-batch repeatability		
	X^a^	SD^b^	CV%^c^	X	SD	CV%
#1	0.789	0.045	5.7	0.756	0.034	4.5
#2	1.057	0.037	3.5	0.844	0.027	3.2
#3	1.167	0.021	1.8	0.830	0.044	5.3
#4	0.760	0.019	2.5	0.630	0.017	2.7
#5	1.065	0.033	3.1	1.150	0.023	2.0

a Mean S/P value

b Standard deviation

c Coefficient of variation

### Evaluation of the diagnostic sensitivity and specificity of I-ELISA

IFA is the gold standard for serological confirmation of PCV-2 infection. Thus, the performance of the indirect ELISA was compared with that of the IFA assay. Among 120 PCV-2-positive samples detected by IFA, 118 samples were tested PCV-2-positive by the I-ELISA (Table 4). On the other hand, among 30 samples PCV-2-sero-negative tested by IFA, 28 samples were PCV-2-negative by this I-ELISA. These results indicated that the DSe of this I-ELISA was 98.33% and DSp 93.33%. Further, the concordance of this I-ELISA to IFA was 98.66%, confirming that ELISA results have a higher consistence with IFA.

**Table 4:** Diagnostic Sensitivity and Diagnostic Specificity of indirect ELISA

		No. of samples with IFA		Total
		Positive	Negative	
PCV2 VLP I-ELISA	Positive	118	2	120
	Negative	2	28	30
	Total	120	30	

### Serological survey of PCV-2-infected pigs using the I-ELISA

Evaluation of the performance of the indirect ELISA was performed using 170 serum samples from three different regions in China. Pigs of these swine farms were vaccinated with an inactivated chimeric PCV-2 vaccine (Sichuan HuaPai Bio-pharmaceutical Co., Ltd., Chengdu, China). With the S/P cut-off value at 0.438 for differentiating PCV-2-positive and -negative serum samples, the results showed that 151 samples were positive (88.9%) and 19 samples (11.1%) were negative for PCV-2 antibodies. Regionally, the positive rate was 97.1% (68/70) in Hunan Province, 80.0% (28/35) in Henan Province and 84.6% (55/65) in Shandong Province (Table 5). These data suggest that this ELISA may act as a rapid, low-cost, reliable and useful tool for the serological evaluation of current PCV-2 vaccine efficacy.

**Table 5:** Prevalence of antibodies to PCV2 in pig sera collected from Henan, Hunan, and Shandong Provinces

Areas	Positive	Negative	Total	Positive rate
Hunan province	68	2	70	97.1%
Henan province	28	7	35	80.0%
Shandong province	55	10	65	84.6%
Total	151	19	170	88.9%

## Discussion

Using recombinant PCV-2 Cap proteins made from insect cells, the established indirect ELISA was with good repeatability, high sensitivity and specificity for detecting PCV-2 antibodies in serum samples. This indirect ELISA was successfully used to detect antibodies in clinical samples from three provinces in China, suggesting that this approach may be used for PCV-2 vaccine evaluation or field infection surveillance.

PCV-2 infection is still being epidemic over the world and causes large economic losses in swine industry [34, 35]. The growing threat of PCV-2 infection to swine health and production necessitates the development of safe and dependable diagnostic assays. ELISA has been used in PCV-2 serological assays and researches have been done on the specificity and sensitivity of PCV-2 ELISA kits [36]. However, although commercial PCV-2 ELISA tests are widely utilized, their high cost impacts the extensive usage in the field. Thus, a novel test with better antigenicity and low-cost is still needed for large-scale surveys.

Recombinant PCV-2 Cap proteins can form VLPs in yeast, insect cell, and *E. coli* expression systems. The VLP morphological structure is similar to that of natural virus particles. It is capable of mimicking external epitopes and conformational epitopes of viruses. Thus, PCV-2 VLPs have been widely used as an antigenic target for PCV-2 infection serodiagnosis in a variety of ELISA formats (indirect and competitive ELISAs) [21–24]. Juozas Nainys et al. demonstrated that recombinant PCV-2 VLPs generated in the yeast Sacharomyces cerevisiae are a suitable substitute for PCV-2 antigen in indirect ELISA for the detection of PCV-2 antibodies in pigs [37]. Shang et al. created an alternative indirect ELISA for the detection of PCV-2 antibodies by using the nuclear localization signal-truncated capsid protein of PCV-2 produced in *E. coli* (CAP ELISA) [32]. The baculovirus insect cells expression system can produce the better antigens for subunit vaccines, the antigenicity of PCV-2 Cap VLPs produced in baculovirus expression system was validated [38, 39]. However, PCV2 VLP produced using baculovirus insect cells expression system used for sero-diagnostic antigens are not reported yet. In this study, the baculovirus expression system has been employed successfully to express and produce PCV-2 VLPs. Using PCV-2 VLPs for coating antigens has advantages, including cost effectiveness, high yield, and better antigenicity.

A good ELISA detection is evaluated by its repeatability, high sensitivity and specificity. To this end, the less coating antigen PCV-2 VLPs (2 μg/mL) was used in this ELISA compared with 4 μg/mL by Jung et al. [40]. Analytical specificity was assessed using cross-reactivity testing, and the findings indicate that I-ELISA is PCV-2 specific. In the present study, the cutoff value calculated from the Mean + 3SD was 0.438 for the indirect ELISA. It is expected to detect every possible case as the cutoff point (0.438) was chosen even though a higher number of false-positive. Further, 170 serum samples from three provinces in China were tested for its validation, and the results were satisfactory when compared to the serological information tested by the commercial ELISA (data not shown). Based on the findings, the established ELISA can be used to identify hotspot regions when the large-scale surveys are undertaken, and may provide an important serological diagnostic method for PCV-2 epidemiology.

In conclusion, we have developed an I-ELISA based on PCV-2 VLPs to detect PCV-2 antibodies with high specificity, sensitivity, and reproducibility. It may have great potential usage for serum epidemiological study and PCV-2 vaccination evaluation as well as the serological diagnosis of PCV-2 infection.

## Electronic supplementary material

Below is the link to the electronic supplementary material.

Supplementary Material 1

Supplementary Material 2

## Data Availability

All relevant data in this study are available from the corresponding author upon reasonable request.
