# Supplementary material for: Production of virus-like particles of porcine circovirus 2 in baculovirus expression system and its application for antibody detection
Source: BMC Vet Res. 2023 Jul 19;19:87. doi: 10.1186/s12917-023-03648-7 (PMC10355036; doi:10.1186/s12917-023-03648-7)
Supplement: Supplementary file 2 — Supplementary Material 2 [file 12917_2023_3648_MOESM2_ESM.pdf]

Production of virus-like particles of porcine circovirus 2 in baculovirus expression system and its application for antibody detection

Yanwei Li, Pingping Yu, Yaxuan Bao, Yuwen Ren, Shaowei Zhao, Xuexian Zhang*

Beijing Kemufeng Biopharmaceutical Co., Ltd, No.25 Xiangrui Street Daxing District, Beijing 102600 China

*Corresponding author：Xuexian Zhang

E-mail：xuexianz@hotmail.com

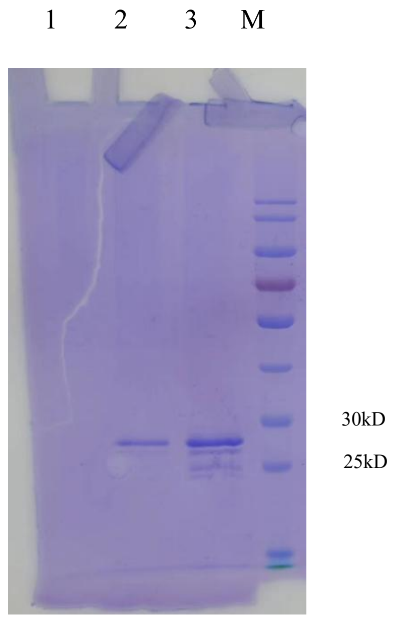

Supp_of Figure 1A Coomassie blue stain shows overexpression of the purified protein. Lanes 1 is uninfected sf9 cell lysates; lanes 2 and 3 are cell lysates infected with pFastBac™Dual plasmid and recombinant baculovirus recombinant baculovirus passage 1, respectively.

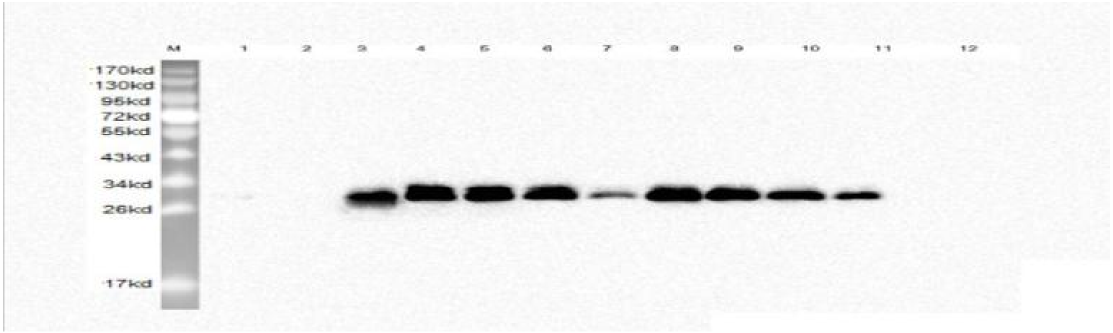

Supp_of Figure 1C Identification of recombinant protein expression by Western blot. Lanes 1 is uninfected Sf9 cell lysate; lanes 2, Sf9 cells infected with pFastBac™Dual plasmid; lanes 3 are sf9 cell lysates at 72 h after infection with recombinant baculovirus passage 1, lanes 4 to 7 are sf9 cell lysates at 56 h, 48h, 33 h and 24 h, Lanes 8 to 11 are hi5 cell lysates at 72 h, 56 h, 48 h and 33 h after infection with recombinant baculovirus passage 1; Lanes 12 is uninfected hi5 cell lysate.
